# The role of TRIM family in metabolic associated fatty liver disease

**DOI:** 10.3389/fendo.2023.1210330

**Published:** 2023-10-05

**Authors:** Jingyue Zhang, Yingming Zhang, Ze Ren, Dongmei Yan, Guiying Li

**Affiliations:** ^1^ Key Laboratory for Molecular Enzymology and Engineering of the Ministry of Education, School of Life Sciences, Jilin University, Changchun, China; ^2^ Department of Immunology, College of Basic Medical Sciences, Jilin University, Changchun, China

**Keywords:** metabolic associated fatty liver disease, TRIM family, insulin resistance, ubiquitination, metabolism

## Abstract

Metabolic associated fatty liver disease (MAFLD) ranks among the most prevalent chronic liver conditions globally. At present, the mechanism of MAFLD has not been fully elucidated. Tripartite motif (TRIM) protein is a kind of protein with E3 ubiquitin ligase activity, which participates in highly diversified cell activities and processes. It not only plays an important role in innate immunity, but also participates in liver steatosis, insulin resistance and other processes. In this review, we focused on the role of TRIM family in metabolic associated fatty liver disease. We also introduced the structure and functions of TRIM proteins. We summarized the TRIM family’s regulation involved in the occurrence and development of metabolic associated fatty liver disease, as well as insulin resistance. We deeply discussed the potential of TRIM proteins as targets for the treatment of metabolic associated fatty liver disease.

## 1 Introduction

Metabolic associated fatty liver disease (MAFLD) refers to fatty liver disease associated with metabolic and cardiovascular abnormalities like obesity, type 2 diabetes mellitus (T2DM), and hypertriglyceridemia. The term MAFLD, proposed by an international expert consensus statement in 2020, previously named to as nonalcoholic fatty liver disease (NAFLD) which refers to the presence of steatosis in more than 5% of the liver cells in non-alcoholic patients (1). There is a significant overlap between NAFLD and MAFLD (2), and findings from NAFLD studies can be basically equivalent to data on MAFLD. In 2023, a multi-society Delphi consensus statement on new fatty liver disease nomenclature suggested that the term metabolic dysfunction-associated steatotic liver disease (MASLD) was chosen to replace NAFLD (3). Recently, the proposed criteria for diagnosing MAFLD require the presence of hepatic steatosis along with at least one of the following conditions: overweight/obesity, T2DM, or indications of metabolic dysregulation (2). As a progressive disease, MAFLD can progress from metabolic overload to persistent hepatocellular injury, and further develop into liver fibrosis, ultimately progressing to cirrhosis or liver cancer (4). MAFLD has emerged as the leading chronic liver disease threatening global human health, with a global morbidity of approximately 25%, and its incidence continues to increase (5). However, the underlying mechanisms of MAFLD remain incompletely understood. In recent years, increasing evidence has shown that endocrine disorders are closely related to MAFLD. The occurrence of MAFLD in turn increases the risk of endocrine-based metabolic dysfunction, such as insulin resistance (IR) and T2DM. The interaction between MAFLD and endocrine disorders may be influenced by multiple factors, including age, hormonal status, metabolic rate, diet, and multiple genetic and epigenetic factors (6).The tripartite motif (TRIM) protein family is an evolutionarily conserved of proteins. It usually has a typical RING finger, B box and coiled-coil (RBCC) domain, and has E3 ubiquitin ligase activity. It has the capacity to mediate protein interactions, engaging in an array of both physiological and pathological mechanisms, such as autophagy, metabolic regulation, immune activation and inflammation (7). Proteins within the TRIM family can facilitate ubiquitination and degradation of various MAFLD-related proteins, thereby playing a pivotal role in the progression of MAFLD (8). In addition, TRIM family proteins are involved in many signaling pathways. The same TRIM protein can directly or indirectly participate in multiple signaling pathways, and TRIM family proteins can also act as MAFLD promoting factor or MAFLD inhibiting factor (9).

This article reviews the role of TRIM family in metabolic-related fatty liver disease. Among them, TRIM8, TRIM11, TRIM15, TRIM24, TRIM25, TRIM26, TRIM31, TRIM37, TRIM38, TRIM39, TRIM50 and TRIM59 are highlighted. We also introduced the structure and function of TRIM protein. This article reviews the TRIM family’s involvement in the occurrence and development of metabolic-related fatty liver disease and the regulation of insulin resistance. We further explored the possibility and limitations of TRIM protein as a potential therapeutic target for the treatment of metabolic-related fatty liver disease, and made a prospect for the future.

## 2 MAFLD

MAFLD ranks as the predominant chronic liver condition globally. NAFLD denotes steatosis in over 5% of hepatocytes, identified through imaging, blood markers, or histology, excluding specific causes like alcohol, viral hepatitis, genetic liver disorders, or extended use of steatogenic medications (10, 11). A liver biopsy remains the definitive test for this diagnosis. The disease can progress from steatosis to conditions like nonalcoholic steatohepatitis (NASH), fibrosis, cirrhosis, and even hepatocellular carcinoma (HCC).

### 2.1 Epidemiology

In the last twenty years, MAFLD has emerged as the leading chronic liver condition globally, affecting an estimated 25.24% of the world’s population. The highest prevalence is observed in the Middle East and South America (12). Previous studies have shown that the risk of chronic liver disease in diabetic patients is about three times higher, mainly related to non-viral and non-alcoholic-related causes, which can be mainly attributed to MAFLD (13). In fact, the prevalence of MAFLD in T2DM patients is more than twice that of the general population, and the incidence is as high as 55.5% (14). A recent meta-analysis revealed that 29.88% of the Chinese population has MAFLD. This prevalence is notably higher in T2DM patients, at 51.83%, compared to 30.76% in non-diabetic individuals. Obese individuals showed a striking rate of 66.21%, while the lean demographic registered at 11.72% (15). The surge in MAFLD in China aligns with the escalating trend of obesity, which saw an increase from around 2% in 2000 to 7% in 2014 (16). Furthermore, T2DM and obesity heighten the progression risk from mere steatosis to conditions like NASH, cirrhosis, and HCC (17, 18). Significantly, China tops the charts in Asia for MAFLD prevalence, morbidity, and annual mortality (19). The global surge in MAFLD is propelled by the rising incidences of type 2 diabetes and obesity.

Metabolic adaptations vary between thin MAFLD and obese MAFLD patients, which might help clarify the pathophysiology of thin MAFLD (20). A comprehensive meta-analysis covering 93 studies from 24 countries/regions revealed that 5.1% of the global population has thin MAFLD, while 12.1% have non-obese MAFLD (21). Significantly, even among those with a regular waist circumference, MAFLD is not rare, with a prevalence of 12.9% (22). Thus, even those of slender or average weight, when coupled with metabolic anomalies, account for a noticeable segment of MAFLD cases.

### 2.2 Pathophysiology

NAFLD can be categorized into two primary types. The first is closely linked to metabolic syndrome, with insulin resistance identified as its principal pathophysiological mechanism. The second type is associated with infectious pathologies leading to liver steatosis. Causes range from viral infections like hepatitis C and HIV to certain medications (including total parenteral nutrition, glucocorticoids, tamoxifen, tetracycline, amiodarone, methotrexate, valproic acid, and vinyl chloride). It’s also connected to specific toxins or hereditary/acquired metabolic conditions such as lipodystrophy, cachexia, and outcomes from procedures like intestinal bypass surgery (23, 24).

### 2.3 Pathogenesis

The pathogenesis of MAFLD arises from intricate interactions among environmental factors, metabolic and demographic variables, genetic differences, and gut microbiota (25). Our current comprehension points to excessive lipid accumulation in the liver. This imbalance is attributed to lifestyle changes linked with high-caloric intake, sedentary behavior, and multiple sclerosis. A diet rich in energy contributes to increased free fatty acid uptake in the liver, augmented lipolysis in adipose tissue, and regeneration of liver fat. Despite mechanisms related to fatty acid oxidation and very low-density lipoprotein (VLDL) secretion, they are inadequate to offset triglyceride accumulation (26). An abundance of liver saturated fatty acids can lead to lipotoxicity and escalate endoplasmic reticulum stress (27, 28), while heightened lipid oxidation intensifies oxidative stress in the liver. Bovi et al. (29) offered an extensive analysis on this topic. Furthermore, the suboptimal diet observed in NAFLD patients might alter microbiota compositions, characterized by diminished short-chain fatty acid (SCFA) production, increased gut permeability, and the translocation of intestinal bacteria or their byproducts to the liver, fostering inflammation. This in turn activates hepatic stellate cells, prompting collagen release and triggering fibrosis, thereby advancing the disease.

## 3 Structure and activity regulation of TRIM family proteins

**Figure 1:**
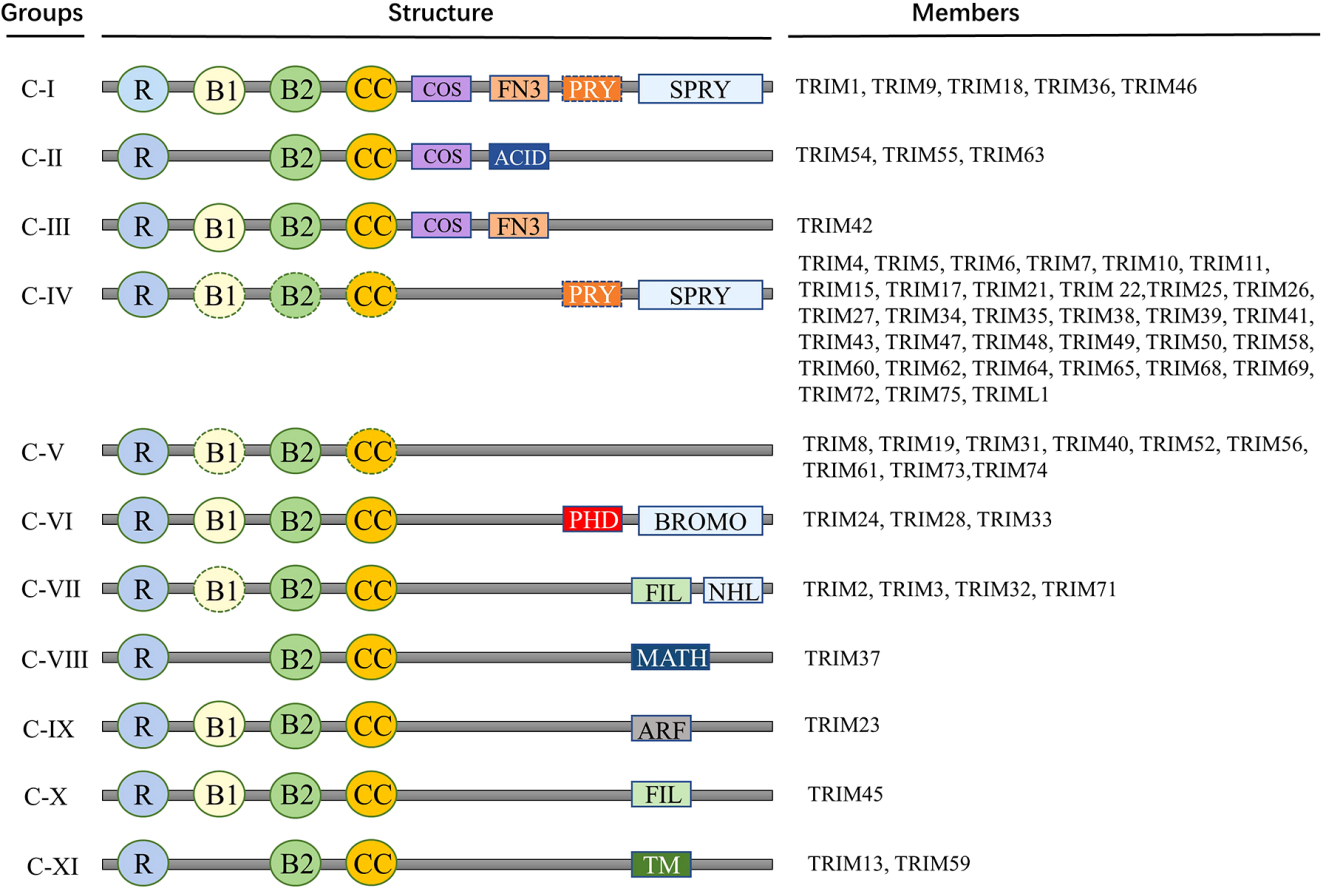
Members and protein structure of TRIM family. Proteins of TRIM family contain more than 80 members. This includes a RING-finger domain, one or two B-box zinc-finger domains, and a coiled-coil domain, collectively forming the RBCC domain specific to the TRIM family. Given the significant variability in the C-terminal domain, TRIM proteins possessing a RING domain can be categorized into 11 subgroups, ranging from C-I to C-XI. R, RING domain; B, B-box domain; CC, coiled-coil domain; COS, Cos-box domain; ACID, Acid domain; FN3, Type III fibronectin repeat sequence; PRY, PRY domain; SPRY, SPRY domain; ARF, ADP-ribosylation factor family domain; BROMO, Bromo domain; FIL, Silk protein immunoglobulin domain; MATH, Meprin and TRAF homologous domains; NHL, NCL1, HT2A and LIN41 domains; TM, Transmembrane domain.

The defining characteristic of the TRIM superfamily is the presence of an N-terminal TRIM motif. This includes a RING-finger domain, one or two B-box zinc-finger domains, and a coiled-coil domain, collectively forming the RBCC domain specific to the TRIM family (30). The RING domain is instrumental in facilitating protein-protein interactions. Most of the TRIM family proteins exhibit ubiquitin E3 ligase activity because they contain a RING-finger domain. RING domain dimerization is usually a prerequisite for ubiquitin ligase activity. B-box domains consist of concise peptide sequences with finger-like extensions, playing a crucial role in recognizing target proteins. Meanwhile, the coiled-coil domain aids in the assembly of TRIM protein homopolymers and heteropolymers (31). In general, the RBCC domain of the TRIM family is highly conserved, so that the major structural differences between members of the TRIM family are determined by additional C-terminal domains of various kinds (32). TRIM proteins are widely expressed in mammalian cells. To date, more than 80 TRIM family proteins have been identified in humans and mice (33). Given the significant variability in the C-terminal domain, TRIM proteins possessing a RING domain can be categorized into 11 subgroups, ranging from C-I to C-XI (**Figure 1**) (30–34). While the arrangement and sequence of domains may vary, resulting in different specialized functions, the intervals between each domain remain consistently conserved. Studies have shown that TRIM family members are involved in a wide variety of cellular activities and processes, such as mRNA localization (35), autophagy (36, 37), cell death, apoptosis (7), cell cycle progression and mitosis (38), DNA damage response (39), viral infection (40), metabolic regulation, immune activation and inflammation (41, 42).

### 3.1 RING domain

Almost all TRIM proteins feature the RING domain at their N-terminus, specifically on amino acid residues 10-20, which houses a zinc-finger motif (43–45). This RING domain, found in numerous TRIM family members such as TRIM5α, TRIM8, TRIM11, TRIM21, TRIM22, and TRIM25, demonstrates E3 ubiquitin ligase activity, facilitating ubiquitination (31, 43–53). The type of polyubiquitination that a TRIM protein binds to dictates the modified target protein’s fate (54–58). For instance, K48-linked ubiquitination triggers proteasomal degradation of the substrate, while K63-linked polyubiquitination typically modifies the substrate’s function, its subcellular positioning, or its protein-protein interaction potential (59, 60).The RING domain’s E3 ubiquitin ligase activity not only facilitates ubiquitin linkage to targets but is also crucial for the anti-HIV capabilities of certain TRIM proteins and in mediating signal transduction after innate immune receptor activation. This domain enables TRIM family proteins to modulate a spectrum of host and viral substrates through ubiquitin or ubiquitin-like (UBL) modifications (61). For instance, TRIM25 can auto-modify and modify other proteins by attaching to ISG15, independent of its RING domain (62, 63). The RING domains in TRIM63 and TRIM19 interact with the SUMO-conjugating enzyme UBE2I, hinting at potential roles in protein SUMOylation (31). Such TRIM proteins can facilitate the binding of UBL proteins, like ISG15 or SUMO, showcasing the RING domain’s enzymatic versatility (64).

### 3.2 B-box domain

TRIM proteins commonly feature one or two B-box domains, each with a zinc-binding motif. These domains are classified as B-box 1 and B-box 2, differing in length and sequence across the TRIM superfamily. Notably, B-box 2 is located downstream of B-box 1, and when only one B-box is present, it’s typically B-box 2 (64). Though the exact function of B-box domains remains elusive, they’ve been associated with regulating TRIM protein assembly, E3 ligase activity, and protein interactions (60). They also play a role in HIV resistance. Specifically, studies on TRIM5α revealed that removing the B-box2 domain impedes the C-terminal PRYSPRY domain’s ability to recognize the viral capsid, diminishing its binding efficacy and compromising TRIM5α’s resistance to viral infections (65–67).

### 3.3 Coiled-coil domain

The coiled-coil domain comes after the B-box in TRIM proteins. It typically mediates TRIM protein homodimerization and, occasionally, heterodimer assembly (68). Enabled by this domain, protein interactions form sizable complex structures (69). It’s especially vital for antiviral TRIM proteins like TRIM5α, where it’s essential for trimerization and its antiviral actions (70, 71). Research also suggests that, during the E3 ligase activation of some TRIM proteins, the coiled-coil and B-box domains form a pocket structure, accommodating chimeric substrate molecules (31). This hints at a joint functional structure between these domains.

TRIM proteins act as E3 ubiquitin ligases, modulating various factors via ubiquitination. For instance, TRIM50 degrades SNAIL through ubiquitination, reversing epithelial-mesenchymal transition (EMT) and resisting anoikis (72). TRIM25 ubiquitinates MTA1 at K98, curbing HCC metastasis. TRIM7’s interaction with SRC affects the src-mTORC1-S6K1 pathway (73), while TRIM31 triggers the degradation of the TSC1-TSC2 complex, overstimulating mTORC1 (74). Liver-targeted trim28 knockout triggers a gender-biased metabolic syndrome through the ERK1/2-MAPK pathway. This knockout activates genes such as FSP27 and Cyp gene clusters, causing primarily male hepatic steatosis and adenomas (75).

## 4 TRIM family and MAFLD

MAFLD refers to a clinicopathological syndrome characterized by diffuse hepatic macrovesicular steatosis, including simple fatty liver and its evolving steatohepatitis, fibrosis and cirrhosis (5). MAFLD is characterized by liver lipid accumulation, accompanied by inflammation and insulin resistance. A large number of patients diagnosed with MAFLD will further develop into steatohepatitis. In severe cases, steatohepatitis will also develop into liver fibrosis, cirrhosis, and even hepatocellular carcinoma, ultimately leading to death. The incidence of MAFLD continues to rise as people’s living conditions improve and their lifestyles change. MAFLD has emerged as the primary driver of chronic liver disease in numerous regions worldwide (76). MAFLD can also lead to a significant burden of extrahepatic chronic complications, which has become a medical and social problem of global concern (7). Current statistics data have shown that MAFLD is more common in men than women, with an incidence of approximately 20 per 10,000 person each year, peaking at the age of 60 years (76). The current prevalence of MAFLD is around 30-40% in men and around 15-20% in women (77), and is even higher in patients with T2DM, with an incidence of up to 70% in this patient group (78). A series of studies in the past have shown that the clinical impact of MAFLD is not limited to liver-related morbidity and mortality, and there is now increasing evidence that MAFLD is a multi-system disease involving multiple extrahepatic organs and regulatory pathways (79). The complex pathogenesis and genetic basis need to be further elucidated to provide a theoretical basis for clinically effective intervention in MAFLD.

### 4.1 TRIM family proteins regulate the occurrence and development of MAFLD

The TRIM family plays pivotal roles in diverse cellular signaling pathways and biological functions. Additionally, its members have been implicated in cancer initiation, progression, and resistance to therapy, displaying both oncogenic and tumor-suppressive capacities depending on the specific type of human cancer. In this review, we delve into the intricate mechanisms through which TRIM family members influence the onset and progression of chronic liver disease and cancer. Hepatocellular steatosis is a key feature of MAFLD, accumulation of fat and the subsequent fibrosis in the liver can escalate the evolution of MAFLD into liver fibrosis, cirrhosis, and even hepatocellular carcinoma.

#### 4.1.1 TRIM and steatohepatitis

Overweight and obesity have a strong pathological association with MAFLD and are one of the key factors in predicting adverse clinical outcomes. Research has indicated that the TRIM family holds a pivotal regulatory function in the process of liver pathology (80). For example, TRIM 8 is almost not expressed in the liver, but can be induced in obese conditions. Increased expression of TRIM8 can activate liver inflammation, disrupt lipid metabolism homeostasis, and promote the progression of obesity-induced MAFLD, while the absence of TRIM8 has a protective effect on these pathophysiological processes (81). A large amount of evidence suggests that MAPK signaling pathway is intimately involved in the initial development of MAFLD and its complications. Multiple upstream signaling molecules including ASK1, transforming growth factor-beta-activated kinase 1(TAK1), MEKK1, can activate JNK and p38 (82). Yan et al. found that only the activity of TAK1 changed with the expression of TRIM8 under high-fat diet (HFD) feeding. The phosphorylation level of TAK1 in TRIM8 overexpression (TRIM8-HTG) mice was markedly elevated compared to the control group, while the phosphorylation level of TAK1 in TRIM8 knockout (TRIM8-HKO) mice was decreased. However, the activity of ASK1 and MEKK1 is not regulated by TRIM8 (83). The above results suggest that TRIM8 may regulate the occurrence of MAFLD by regulating JNK/P38 signaling pathway through TAK1.

Current studies have shown that TRIM59 is highly expressed in MAFLD tissues (84). It was observed that TRIM6, TRIM9, TRIM22, TRIM59 and TRIM69 exhibited higher expression in severe MAFLD tissues compared to those with mild MAFLD. Relative to normal liver tissues, TRIM59 expression in MAFLD tissues showed a significant elevation, with the most pronounced increase observed in severe MAFLD samples. The results showed that TRIM59 mRNA level was positively correlated with the progression of MAFLD. In addition, the level of triglyceride, Fe^2+^ and ferritin in MAFLD tissues was notably elevated compared to those in normal tissues, and the highest in severe MAFLD tissues. Palmitic acid (PA) is a fat-producing agent that is routinely used to induce steatosis in cultured hepatocytes. In L02 cells, PA treatment notably intensified steatosis levels, elevated TRIM59 expression, and enhanced the secretion of tumor necrosis factor alpha (TNF-α), interleukin 6 (IL-6), and interleukin 8 (IL-8). Amplifying the expression of TRIM59 further exacerbated steatosis and substantially heightened the secretion of TNF-α, IL-6 and IL-8. These findings suggest that TRIM59 can amplify PA-induced steatosis and inflammation in MAFLD cell models (84).

The application of ferroptosis inhibitor defetoxamine (DFO) can significantly improve TRIM59-mediated steatosis and inflammation. Overexpression of TRIM59 can promote the steatosis of L02 cells, and the application of DFO can significantly reverse this effect (85). At the same time, TNF-α and over expression of TRIM59 could significantly promote the expression of IL-6 and IL-8, while DFO could apparently reduce the expression of IL-8. Flow cytometry showed that TRIM59 could significantly increase lipid ROS levels, and DFO can significantly reduced lipid ROS levels. In addition, TRIM59 has been demonstrated to interact with glutathione peroxidase 4 (GPX4) and facilitate its ubiquitination. GPX4 is a pivotal enzyme that shields cells from lipid peroxidation. Concurrently, suppressing GPX4 can lead to ferroptosis (86). A previous study showed that elevated GPX4 levels were associated with reduced severity of MAFLD (87). Overexpression of TRIM59 could down-regulate the GPX4 protein levels without influencing its mRNA level. This suggests that TRIM59 may modulate the post-transcriptional modification of GPX4. Further analysis showed that TRIM59 promoted its ubiquitination by interacting with GPX4, thereby enhancing the proteasome degradation of GPX4 to down-regulate its protein level and induce MAFLD. Overexpression of GPX4 can significantly reverse the pathogenic role of TRIM59 in MAFLD. In conclusion, TRIM59 can promote MAFLD steatosis and ferroptosis by enhancing GPX4 ubiquitination, and inhibiting TRIM59 can effectively improve the MAFLD process, so TRIM59 may be a potential target for MAFLD treatment (84).

#### 4.1.2 TRIM and hepatic fibrosis

Chronic liver injury produces an array of inflammatory mediators that activate hepatic stellate cells (HSCs), transforming them into myofibroblasts. These myofibroblasts persistently secrete collagen types I and III, along with other components of the extracellular matrix, leading to the progression of liver fibrosis. As of now, fibrosis remains a condition without an effective treatment (88). However, recent insights into the pathological mechanisms of fibrosis suggest that ubiquitination might be a promising therapeutic target. Two primary pathways mediate protein degradation, with ubiquitin proteolysis being one of them. This pathway oversees the degradation of a significant portion of intracellular proteins. The process of ubiquitination is orchestrated through a cascade of ubiquitin-activating enzymes (E1), ubiquitin-conjugating enzymes (E2) and ubiquitin ligases (E3). Notably, the TRIM family stands as one of the most expansive E3 ligase families, boasting over 80 identified members, the majority of which possess E3 ubiquitin ligase activity. This family is multifaceted, influencing cellular activities ranging from inflammation, immunity, and carcinogenesis to apoptosis, autophagy, and metabolism (9). A growing body of research is now dedicated to unraveling the connections between TRIM and fibrosis.

TRIM8 is a widely expressed E3 ubiquitin ligase, is deeply implicated in cellular processes such as differentiation, the cell cycle, the innate immune response, and apoptosis (80, 89–91). Notably, in contexts of HFD-induced or genetic deficiency (ob/ob)-triggered fibrosis, insulin resistance, inflammation, and hepatic lipid accumulation, TRIM8 binds to and ubiquitinates TAK1. This interaction stimulates TAK1’s autophosphorylation, leading to an intensified activation of downstream JNK/p38, IKKβ-NF-κB, and IRS1-AKT signaling pathways. This escalation in signaling accelerates the progression of liver fibrosis and its associated complications. Strategies that interrupt the TRIM8-TAK1 association or target the E3 ligase activity of TRIM8 have been proven as potent countermeasures against liver fibrosis and metabolic syndrome (83). TRIM24 (also known as TIF1α) serves dual roles: it acts as an E3 ubiquitin ligase targeting p53 for degradation and functions as a nuclear receptor coregulator (92, 93). Mice with liver-specific TRIM24 deletions exhibited disrupted liver homeostasis, spontaneously developing liver fibrosis and hepatocellular carcinoma (94). TRIM38 stands as a potent regulator against lipid accumulation both *in vitro* and in mouse liver studies. Hepatocytes lacking TRIM38 show heightened susceptibility to steatosis, insulin resistance, inflammation, and liver fibrosis. Notably, TRIM38 binds directly to TAB2, facilitating its protein degradation. This action on TAB2, which recognizes K63-linked ubiquitin chains of RIP1 in TNF-α signaling or TRAF6 in IL-1β signaling, consequently inhibits the TAK1-MAPK cascade, a pathway critical in liver fibrosis progression (95, 96). A central driver of liver fibrosis is the activation of HSCs. In fibrotic liver tissues, TRIM26 expression is suppressed. However, its overexpression results in lipid ROS accumulation, initiating ferroptosis in HSCs. The process is driven by TRIM26-mediated ubiquitination and degradation of SLC7A11, making TRIM26 a promising therapeutic agent against liver fibrosis (97). Additionally, both TRIM15 is recognized for their ability to suppress HSC activation, subsequently mitigating liver fibrosis (98, 99).

#### 4.1.3 TRIM and liver cancer

Empirical research underscores a profound association between the TRIM family and HCC. These proteins demonstrate a diverse spectrum of functions throughout various HCC developmental processes. Intriguingly, individual TRIM family members manifest distinct roles in HCC, exhibiting both oncogenic and tumor-suppressive effects. Remarkably, certain TRIM proteins can assume opposing roles during different phases of liver cancer progression (100, 101). In specific scenarios, TRIM expression levels might serve as diagnostic biomarkers and prognostic indicators for cancer.

The Keap1-Nrf2 pathway is recognized as the primary signaling mechanism governing cellular defenses against oxidative stress (102). Among genes responsive to ER (endoplasmic reticulum) stress, TRIM25 emerges as the most significantly upregulated. This gene is crucial for tumor cell resistance against ER stress, operating as a feedback mechanism. Diverse functional investigations reveal that, in multiple cancer cell models, TRIM25 fortifies tumor cell survival. It achieves this by directing the ubiquitination and subsequent degradation of Keap1, thereby stimulating Nrf2 signaling and curtailing ROS levels during endoplasmic reticulum stress (103). Specifically in HCC, TRIM25 enlivens Nrf2 through the targeted ubiquitination and degradation of Keap1. This action suppresses ROS generation during endoplasmic reticulum stress, bolsters antioxidant defenses, and advances cell survival. Therapeutically, targeting Nrf2 might offer potent interventions against certain cancers. Moreover, TRIM11 and TRIM21 both influence redox homeostasis through distinct pathways, a mechanism integrally tied to tumor onset and progression (104). Diminished TRIM26 expression in tumor specimens significantly correlates with a bleak prognosis for HCC patients. TRIM26 suppression augments HCC cell growth attributes, such as proliferation, colony formation, migration, and invasion. Bioinformatic assessments pinpoint TRIM26’s role in HCC cell metabolism modulation. It possibly orchestrates key metabolic entities like ubiquitin-specific protease 2 (USP2) and ATP-citrate lyase (ACLY), either directly or indirectly, exerting a profound influence on mitochondrial metabolic functions (105). In aggressive liver cancer forms, heightened aerobic glycolysis may amplify tumorigenic potentials. Data from The Cancer Genome Atlas (TCGA) indicates elevated TRIM37 expression in HCC tissues compared to their normal counterparts. TRIM37 accentuates the malignant characteristics of HCC by liaising with the p53 protein, invoking E3 ligase activity, promoting ubiquitination, degradation, and favoring the glycolytic pathway. Neutralizing p53 effectively counters TRIM37’s promotive impact on HCC cell growth and metastasis (106). TRIM29 has connections to a diverse range of human malignancies. Its overexpression in lung, gastric, and ovarian cancers correlate with tumor advancement and an unfavorable prognosis (107–109). Conversely, in prostate and breast cancers, TRIM29 exerts a suppressive influence (110, 111). The Wnt/β-catenin signaling pathway is recognized for its pivotal role in the genesis and progression of numerous tumors (112). Notably, silencing TRIM29 in HCC cells activates this pathway, suggesting that TRIM29 might counteract HCC progression by inhibiting the Wnt/β-catenin pathway (113). TRIM50, a more recently confirmed member of the TRIM family, has been revealed to suppress tumor growth in HCC. It achieves this by directly targeting the protein SNAIL, reversing the EMT process (71). TRIM59 emerges as a promising multi-cancer biomarker with potential synergistic implications (114). Valiyeva et al. identified heightened TRIM59 expression in the cytoplasm across 37 tumor types out of 291 human cancer instances (115). In the context of hepatocellular carcinoma, EMT significantly impacts tumor invasion and metastasis. The diminished presence of the epithelial marker, E-Cadherin, is often perceived as an EMT indicator (116). TRIM59 silencing markedly reduces E-cadherin expression while amplifying mesenchymal markers like N-cadherin and vimentin. Conversely, enhanced TRIM59 expression boosts E-cadherin levels while repressing N-cadherin and vimentin. This suggests that TRIM59 augments liver cancer cell EMT by upregulating genes inducing EMT, thereby intensifying the migratory and invasive capabilities of these cells (117).

As E3 ligases central to protein ubiquitination modifications, the TRIM family has emerged as a key player in the genesis and progression of diverse liver ailments. Crucial signaling pathways, including Keap1-Nrf2, Wnt/β-catenin, and NF-κB, which are modulated by TRIM proteins, are intimately linked to the steatohepatitis, liver fibrosis, and hepatocellular carcinoma (118). This underscores the potential of TRIM as a promising target for addressing chronic liver conditions and liver cancer.

### 4.2 Effect of TRIM family proteins on hepatic insulin resistance

Increasing evidence has shown that MAFLD is associated with a variety of insulin-dependent metabolic damage, and patients with type 2 diabetes have significantly higher insulin resistance index. Liver steatosis caused by various reasons, further adipogenic changes, liver dysfunction, and inflammatory damage in the liver can induce and aggravate insulin resistance (119). Insulin resistance index is the strongest predictor of liver inflammation and fibrosis in patients with MAFLD (120). Insulin resistance is often caused by cytokines (TNF-α/IL-6), ER stress activation and JNK activation (121). Aberrant regulation of hepatic glucose and lipid production mediated by insulin is a pivotal event in the pathogenesis and development of MAFLD (122). In healthy individuals, insulin inhibits gluconeogenesis while promoting adipogenesis. In MAFLD, especially in the case of T2DM, IR leads to a decrease in the ability to inhibit gluconeogenesis, while insulin-driven adipogenesis still exists or even increases. Research using animal models has revealed that a deficiency in hepatic Akt signaling results in augmented gluconeogenesis. Additionally, impaired activation of protein kinase C (PKC) λ correlates with reduced blood lipids and diminished expression of sterol regulatory element binding protein-1c (SREBP1c) (123).

Recent data suggest that lipid metabolism itself may also trigger insulin resistance. Diacylglycerol (DAG) is an intermediate product in the synthesis of triglycerides (TAG) from free fatty acids (FFA). Its accumulation in hepatocytes leads to an increase in the amount of PKCϵ transferred to the cell membrane in the liver. PKCϵ binds to insulin receptors on the plasma membrane and inhibits its intracellular kinase domain (124, 125), thereby weakening the insulin signaling pathway and producing insulin resistance. In a recent study, patients with obesity but no symptoms of diabetes were investigated and it was found that DAG content and PKCϵ activation were the most significant predictors of hepatic insulin resistance and it could explain that 60% of the differences in hepatic insulin sensitivity (126).

Recent studies have revealed that TRIM8 aggravates hepatic insulin resistance (127). Using hepatocyte-specific TRIM8 overexpression (TRIM8-HTG) and knockout (TRIM8-HKO) mice subjected to a high-fat diet, the study observed increased body weight, and elevated fasting blood glucose and insulin levels in both non-transgenic (NTG) and TRIM8-HTG mice compared to those on a standard diet. Under HFD, the TRIM8-HTG mice showed a more pronounced increase in fasting blood glucose and insulin levels than controls, though body weight remained similar. In glucose and insulin tolerance tests, TRIM8-HTG mice consistently exhibited higher blood glucose levels than their control counterparts. By further detecting the expression and phosphorylation levels of insulin signaling pathway and related molecules, the results showed that TRIM8 liver-specific overexpression could significantly weaken p-IRS1Tyr608, p-AKTSer473 and p-GSK3β, and significantly reduce the expression of PEPCK and G6Pase. Contrary to TRIM8-HTG mice, hepatocyte conditional knockout TRIM8-HKO mice showed a reduced phenotype after HFD feeding. Taken together, these results indicate that upregulation of TRIM8 expression aggravates hepatic insulin resistance induced by high-fat diet feeding.

### 4.3 TRIM regulates the interaction between MAFLD and insulin resistance

Insulin affects the adipogenesis signaling pathway, the adipogenesis transcription factors liver X receptor (LXR), SREBP1c and upstream stimulator-1 (Usf-1), and then regulates insulin-related signaling pathways such as PTPN1 and SOCS-3-STAT to participate in the occurrence and development of MAFLD (128). Wu et al. (129) discovered that heightened expression of miR-206 can modulate the activation of PTPN1-mediated insulin signaling pathway. This modification leads to the suppression of Sbrep1c transcription, an enhancement in glycolysis, and a decrease in lipid buildup within hepatocytes. Tan et al. (130) have shown that hepatocyte nuclear factor 1α (HNF1α) regulates SOCS-3-STAT3 signaling pathway to participate in insulin resistance, affects hepatocyte lipid metabolism and free fatty acid-induced hepatocyte steatosis. The expression of HNF1α is downregulated in hepatocytes with excessive lipid accumulation, which aggravates the lipid degeneration of hepatocytes, while the up-regulated expression of HNF1α can activate the insulin signaling pathway to promote the oxidative degradation of fat and reduce lipid synthesis.

Drugs that regulate insulin-related signaling pathways have been used to treat MAFLD. GLP-1 receptor agonist, liraglutide, can effectively treat MAFLD. Studies have shown that liraglutide up-regulates the expression of adenylate cyclase 3, activates the cAMP/PKA/STAT3 signaling pathway, stimulates Kupffer cells M2 polarization, and reduces hepatic steatosis (131). In addition, Yang et al. (132) found that liraglutide up-regulated the expression of InsR/IGF-1R and IRS2, and activated PI3K/Akt to reduce hepatic steatosis. Liraglutide can also activate the SHP1/AMPK signaling pathway to promote lipid metabolism, inhibit hepatic steatosis, and protect hepatocytes (133).

Yan et al. (127) found that TRIM8 could not only aggravate insulin resistance induced by high-fat diet feeding, but also increase lipid accumulation in the liver and cause liver steatosis. Consistent with the insulin resistance function, TAK1 specific inhibitor 5Z-7-OX almost completely offset the positive regulation of TRIM8 on liver weight gain, lipid accumulation and abnormal liver function. The use of 5Z-7-OX significantly reduced the activity of JNK and IKKβ related to inflammation, and increased the phosphorylation level of AKT in insulin signaling pathway, suggesting that insulin sensitivity was enhanced.

Studies have further clarified the mechanism of TRIM8 promoting insulin resistance and NASH (134). The authors found that TRIM8 overexpression significantly increased the phosphorylation levels of JNK and p38 proteins, but had no effect on ERK activation. Detection of upstream kinases in the MAPK signaling pathway showed that the activity of TAK1 but not MEKK1 or ASK1 could be significantly altered by TRIM8. At the same time, TAK1 specific inhibitor 5Z-7-OX completely blocked the effect of TRIM8 on HFD-induced insulin resistance. Most importantly, 5Z-7-OX almost completely reversed the promoting effect of TRIM8 on liver weight, lipid accumulation, inflammatory response and abnormal liver function. Studies have shown that TRIM8 promotes HFD-induced insulin resistance and NASH by activating JNK and p38 through TAK1.

It has been reported that the phosphorylation and activity of TAK1 are largely dependent on its own ubiquitination (31, 134). Some researchers have explained the molecular mechanism by which TRIM8 interacts with TAK1 to promote TAK1 ubiquitination (31). It was found that palmitic acid stimulation promoted the translocation of TRIM8 from the nucleus to the cytoplasm and co-localization with TAK1. The deletion of TRIM8 significantly reduced the ubiquitination level of TAK1 and the overexpression of TRIM8 increased the polyubiquitination level of TAK1. In addition, and overexpression of TRIM8 in 293T cells significantly enhanced the polyubiquitination level of TAK1. The TRIM8 (C15A; C18A) mutant deleting its E3 ligase activity lost the activity of ubiquitinated TAK1. In addition, TRIM8 mutant deleting the amino acid residues 59-182, which mediates the interaction with TAK1, also resulted in the loss of TAK1 ubiquitination. Most importantly, interfering with the interaction between TRIM8 and TAK1 can block the effect of TRIM8 on TAK1 ubiquitination and completely reverse the effect of TRIM8 overexpression on HFD-induced insulin resistance and MAFLD phenotype. The results elucidate that the molecular mechanism of TIRM8 interacting with TAK1 and ubiquitinating TAK1 to enhance HFD-induced insulin resistance and MAFLD.

However, studies have shown that TRIM family members can also inhibit the occurrence of MAFLD, such as TRIM31 facilitates the degradation of Rhbdf2 through k48-linked polyubiquitination, This action inhibits the Rhbdf2-MAP3K7 signaling pathway and downstream events mitigating insulin resistance, liver steatosis, inflammation and liver fibrosis caused by genetic factors and high-caloric diets (135). The findings from Xu et al. further demonstrate that that TRIM31 acts as a critical suppressor of MAFLD/NASH and metabolic disturbances, positioning it as a potential molecular target for treating these conditions (135).

## 5 Conclusion

In recent years, many literatures have reported that metabolic diseases such as insulin resistance are closely associated with MAFLD. MAFLD is not only associated with an increased incidence of cirrhosis and hepatocellular carcinoma, but may also be associated with risk factors for extrahepatic diseases such as T2DM and cardiovascular diseases. At present, the mechanism of MAFLD has not been fully elucidated. Studies have found that MAFLD is related to insulin resistance (136, 137). The gradual accumulation of fat in the liver of MAFLD patients leads to hepatocyte degeneration and abnormal liver function. At the same time, when there is a large amount of fat accumulation, the liver glycogen is enhanced. Due to abnormal hepatic fat metabolism, the conversion of glucose to fat is blocked, resulting in increased blood glucose and the formation of T2DM (138, 139). Insulin resistance enhances the lipolysis of triglycerides in adipose tissue through hormone-sensitive lipase, resulting in a large accumulation of free fatty acids in the liver (140). Therefore, T2DM can also lead to the deterioration of MAFLD, aggravate the progression of NASH and fibrosis, and increase the incidence of hepatocellular carcinoma. TRIM38 plays a crucial role in regulating lipid accumulation in hepatocytes both *in vitro* and in mouse liver. Its absence in hepatocytes intensifies steatosis, insulin resistance, inflammation, and liver fibrosis. Thus, TRIM38 emerges as a potential inhibitor in NAFLD’s progression, making it a promising target for therapeutic interventions (96).

As an E3 ubiquitin ligase TRIM family protein, the mechanism of its members has been explored in many fields and has been proved to play a key role in the occurrence and development of nonalcoholic fatty liver disease (**Table 1**). TRIM family proteins are involved in many signaling pathways related to metabolic-related fatty liver disease, such as TGF-β, MAPK and NF-κB. TRIM8 may regulate the occurrence of MAFLD by regulating the JNK/P38 signaling pathway through TAK1 (127). TRIM59 promotes steatosis and ferroptosis in MAFLD by enhancing GPX4 ubiquitination, and inhibition of TRIM59 can effectively improve the process of MAFLD (87). TRIM38 directly binds to TAB2, and recognizes the K63 of RIP1 in the TNF-α signaling pathway through TAB2 to connect the ubiquitin chain or TRAF6 in the IL-1β signaling pathway, thereby inhibiting the key pathway TAK1-MAPK cascade in the process of liver fibrosis (96). Therefore, MAFLD can be treated by studying targeted drugs of TRIM family members such as TRIM8, TRIM11, TRIM15, TRIM24, TRIM25, TRIM26, TRIM31, TRIM37, TRIM38, TRIM39, TRIM50 and TRIM59. Although the epidemiology, pathophysiology and pathogenesis of nonalcoholic fatty liver disease have been extensively studied, the underlying mechanisms remain unclear. Therefore, the development of these targeted drugs requires more in-depth study of their mechanisms. TRIM67 is almost not expressed in the liver, but in the case of obesity, it is inducible, activating liver inflammation, lipid metabolism disorders, and promoting obesity-induced MAFLD (141). However, the specific mechanism remains to be studied. At present, some small molecule inhibitors of TRIM family proteins have been gradually discovered. Studies have found that TRIM31 is a key inhibitor of MAFLD and metabolic disorders. Drugs that regulate insulin signaling-related signaling pathways have also been used to treat MAFLD, but there are still some limitations. For example, it is used in the treatment of liver disease, but the mechanism of adverse reactions to other organs of the body remains to be studied. We are convinced that with the further exploration of the mechanism of the occurrence and development of liver diseases and the development of new targeted drugs, these limitations will be resolved. In the future, targeted drugs for these TRIM family proteins will bring new hope to patients with liver diseases. It is suggested that the regulation of TRIM family on MAFLD is diverse, complex and heterogeneous, which needs to be further studied to provide experimental basis for the effective prevention and treatment of MAFLD.

**Table 1:** The role of TRIM family proteins in the development of MAFLD.

Tripartite motif family protein	Involved signaling pathways	leading role	reference
TRIM 8	MAPK signaling pathway	Regulate the occurrence of MAFLD by regulating JNK/P38 signaling pathway through TAK1	(82)
TRIM11	Keap1-Nrf2 pathway	TRIM11 suppresses tumourigenesis by affecting redox homeostasis	(48)
TRIM15	FAK-related pathway	localizes to focal adhesions due to its interaction with paxillin and regulates focal adhesion turnover	(98)
TRIM24		E3 ubiquitin ligase targeting p53 for degradation and functions as a nuclear receptor co-regulator	(92)
TRIM25	Keap1-Nrf2 pathway	In tumour,TRIM25 directly targets Keap1 by ubiquitination and degradation. This leads to Nrf2 activation, which bolsters anti-oxidant defense and cell survival.	(103)
TRIM26	Xc− antiporter system	Mitigate Liver Fibrosis Through Mediating SLC7A11 Ubiquitination	(97)
TRIM29	Wnt/β-catenin signaling pathway	In hepatocellular carcinoma, depletion of TRIM29 may promote *in vitro* proliferation, clone formation, migration and invasion of hepatocellular carcinoma cells through the Wnt/β-linker protein signalling pathway	(113)
TRIM31	Rhbdf2–MAP3K7 axis	Trim31 alleviates non-alcoholic fatty liver disease by targeting Rhbdf2 in mouse hepatocytes	(135)
TRIM37	TRIM37-P53 axis	Downregulation of TRIM37 destabilises the p53 protein, which in turn promotes aerobic glycolysis and ultimately supports the development of hepatocellular carcinoma	(106)
TRIM38	NF-κB signaling pathway	Inhibits TNFα- and IL-1β–triggered NF-κB activation by mediating lysosome-dependent degradation of TAB2/3	(95)

## Author contributions

JZ, YZ performed data collection and analysis, wrote and revised the manuscript. JZ, YZ, and ZR interpreted the data, and wrote the manuscript. DY supervised the process and revised the manuscript. GL contributed to the review conception and design, wrote and revised the manuscript. All authors contributed to the article and approved the submitted version.
